# Photoacoustic Sounds from Meteors

**DOI:** 10.1038/srep41251

**Published:** 2017-02-01

**Authors:** Richard Spalding, John Tencer, William Sweatt, Benjamin Conley, Roy Hogan, Mark Boslough, GiGi Gonzales, Pavel Spurný

**Affiliations:** 1 Sandia National Laboratories, Albuquerque, NM, USA; 2 Astronomical Institute, Czech Academy of Sciences, Ondřejov, Czech Republic

## Abstract

Concurrent sound associated with very bright meteors manifests as popping, hissing, and faint rustling sounds occurring simultaneously with the arrival of light from meteors. Numerous instances have been documented with −11 to −13 brightness. These sounds cannot be attributed to direct acoustic propagation from the upper atmosphere for which travel time would be several minutes. Concurrent sounds must be associated with some form of electromagnetic energy generated by the meteor, propagated to the vicinity of the observer, and transduced into acoustic waves. Previously, energy propagated from meteors was assumed to be RF emissions. This has not been well validated experimentally. Herein we describe experimental results and numerical models in support of photoacoustic coupling as the mechanism. Recent photometric measurements of fireballs reveal strong millisecond flares and significant brightness oscillations at frequencies ≥40 Hz. Strongly modulated light at these frequencies with sufficient intensity can create concurrent sounds through radiative heating of common dielectric materials like hair, clothing, and leaves. This heating produces small pressure oscillations in the air contacting the absorbers. Calculations show that −12 brightness meteors can generate audible sound at ~25 dB SPL. The photoacoustic hypothesis provides an alternative explanation for this longstanding mystery about generation of concurrent sounds by fireballs.

Concurrent sound associated with very bright meteors manifests itself as popping, hissing, and faint rustling sounds occurring simultaneously with the arrival of the light from the meteor^1^,^2^,^3^,^4^,^5^,^6^,^7^. Concurrent sound occasionally is generated by fireballs^8^ with apparent magnitude (visual brightness) as low^8^,^9^ as *−9,* and numerous occurrences have been documented^1^,^2^ with apparent magnitudes of −*11* to −*13.* These sounds cannot be attributed to direct acoustic propagation from the upper atmosphere for which the travel time would be several minutes. Concurrent sounds must be associated with some form of electromagnetic energy generated by the meteor, propagated to the vicinity of the observer, and transduced into acoustic waves. Prior to now, the means by which energy from meteors could be propagated to Earth and then converted into audible sound has not been adequately explained and validated by experiment. Here we present observational data, experimental results, and numerical models in support of photoacoustic coupling as the mechanism. Recent photometric measurements of fireballs reveal strong millisecond flares and significant brightness oscillations at frequencies of 40 Hz and higher^7^,^8^. Experiments and models show that strongly modulated light at these frequencies and light intensity on Earth from −12 apparent magnitude meteors (same as full moon illumination ~10^−3^ W/m^2^) can radiatively heat common dielectric materials like hair, cloth, paint, etc. This heating can produce small pressure oscillations in the air adjacent to the absorber. These can be loud enough to be audible (~25 dB SPL). A previous hypothesis of coupling to natural antennas from RF radiation generated by plasma oscillations^1^,^2^ does not seem to be adequately supported by observational evidence of radio waves emanating from meteors^12^,^13^,^14^,^15^. The photoacoustic hypothesis seems to better explain this longstanding mystery about the generation of concurrent sounds by fireballs. However, it is possible that both mechanisms contribute to the observed audio signal.

Strong, millisecond-duration flares have been recorded in nearly all bolides observed by the Czech Fireball Network^10^,^11^. The meteors of interest typically have initial speeds below 40 km/s and burn durations longer than 2 s. These optical pulse trains, if converted to sound, often have time characteristics consistent with the popping, swishing, or sizzling noises reported by observers^1^,^2^,^3^. We suggest that each pulse of light can heat the surfaces of natural dielectric transducers. The surfaces rapidly warm and conduct heat into the nearby air, generating pressure waves. A succession of light-pulse-produced pressure waves can then manifest as sound to a nearby observer.

The photoacoustic effect was observed in 1880 by Alexander Graham Bell and colleagues who heard a tone when they illuminated certain dielectric materials with sunlight modulated with a chopper wheel^16^. In 1976 Rosencwaig & Gersho invented Photo-Acoustic Spectroscopy^17^ (PAS) and provided the first detailed understanding of the physics.

For fireballs, the sound pressure waves track the time history of the illumination, and the amplitude depend on the irradiance. Also important to the generation of sound are the thermal conductivity, specific heat, and density of both the dielectric solid and the air as well as the light penetration depth into the solid.

Figure 1a is an open-shutter photograph of fireball EN091214 taken December 9, 2014. Figure 1b is its intensity-time history as recorded by the Czech Fireball Network^18^. The fireball’s average apparent magnitude was reported as -*15*, about ten times brighter than the full moon. Concurrent sounds from this early-evening fireball were heard by people in several nearby locations. Figure 1c shows the Fourier transform of the light intensity, along with the normalized sensitivity of the human ear. We plot these curves together to show that the observer’s hearing is most sensitive above a few hundred Hertz while the signal from the fireball light is maximized below 100 Hz. Despite this mismatch, photoacoustic sound from fireballs is occasionally heard.

## Estimating the Irradiance

Estimating the irradiance on the ground of the EN091214 fireball is instructive. The temperature of the fireball is similar to that of the sun. Thus the ratio of the irradiances of the fireball and the sun should be proportional to the ratio of their visual brightness. The sun’s average magnitude is −26.7, and its irradiance on Earth is E_sun_ ~ 1100 W/m^2^. The magnitude of EN091214 is -15. Therefore, the irradiance on Earth due to the fireball is approximately:


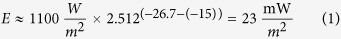


Based on our experiments and simulations, typical dielectric materials change a small fraction (~7 × 10^−8^) of the irradiance (in W/m^2^) on the sample into sound for ***~**1* *kHz* frequencies. The intensity oscillations of the EN091214 fireball are about 1/3^rd^ of the total irradiance which must be included in calculations of the sound pressure level; which is approximately:


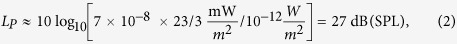


where L_P_ = 1 dB for a sound intensity of 10^−12^ W/m^2^. We estimate that this sound level calculation is accurate to ±3 dB. It is similar in loudness to rustling leaves or faint whispers and is consistent with observations.

## Methods

During our testing we found that the most efficient light-to-sound transducer materials have high absorption coefficients, so the light is absorbed near the surface. They also have low thermal inertia characterized by low conductivity, which minimizes heat flow, and low volumetric heat capacity, which maximizes the temperature rise. This combination of properties is found in most dark-colored dielectric materials. Likely candidates for producing photoacoustic sound are dark paint, fine hair, leaves, grass, and dark clothing – all of which we tested.

Our test setup consisted of a 10 cm square white-light LED array producing a peak flux of E = 5 W/m^2^ on the test sample, the sample, and a scientific grade laboratory microphone. The setup was placed inside a plastic dome located in an anechoic chamber. Outside, we located a signal generator and linear amplifier to drive the LEDs and a spectrum analyzer to record the signal from the microphone.

## Background Calculations

As we begin our analysis of photoacoustic transducers, we note that they tend to fall into two general categories:

- “Half-space” transducers include dark wood, asphalt, and dark paint (the substrate has little effect for thick paint, Δz > 100 μm).
- “Fibrous” transducers include hair, dark clothing, pine-needles, and dry leaves.

**Figure 2:**
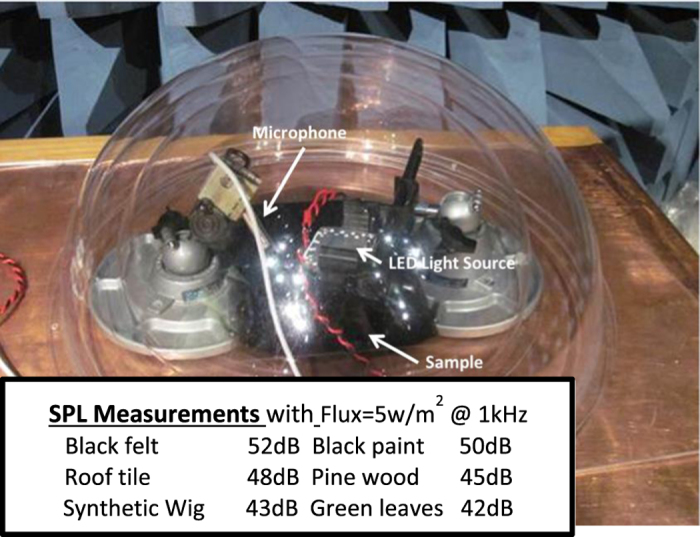
Measurement setup and a list of the Sound Pressure Levels for an irradiance of 5 W/m^2^ at 1000 Hz.

For our analyses we were unable to find properties for many of the materials of interest and therefore had to use proxies. For example, the chemistry and microstructure of leaves, grass and cotton are similar to white pine for which thermal properties are available. Likewise, the composition and structure of hair is similar to leather. Finally, thermal properties of the fibers in synthetic clothing should be similar to those of polyethylene. The average density, specific heat, and thermal conductance of each of these materials can be combined to give typical thermal diffusivities, which for wood, leather, polyethylene, and paint are 0.12, 0.07, 0.18, and 0.28 (μW/(Km^2^)) respectively. These values are similar in magnitude on a log scale, so their “photoacoustic transduction” should also be similar. The experimental results in Fig. 2 tend to validate this statement.

## The Half-Space

Computer simulations were consistent with the experimental results. We used a finite volume analysis to model our half-space with thermal properties of wood. At the surface of the half-space, the calculated amplitude of the temperature variation ΔT (in °C) and the resulting sound pressure level SPL are generated. The following equations have been fitted to the simulated output.


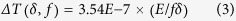



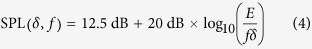


The light penetration depth δ is measured in meters, frequency *f* in Hertz, and incident light flux E(*f*) in W/m^2^. Note that sound pressure levels less than zero dB will not be audible. The following assumptions also apply:

The temperature of the solid is essentially independent of the thermal response of the air due to air’s low thermal conductivity and specific heat. As an approximation, we use a one-way coupled model simulating the solid with a simple Robin boundary condition at the solid-air interface. We first calculate the temperature profile within the solid and then compute the air temperature and pressure fluctuations driven by the oscillating surface temperature.

We assume that the light incident on the solid is spatially uniform and that it varies sinusoidally in time. We specify the light penetration depth δ to be an independent parameter. The light intensity is almost always small, and typically of order 0.01 W/m^2^, so the temperature variations are small and the problem is linear.

**Figure 3:**
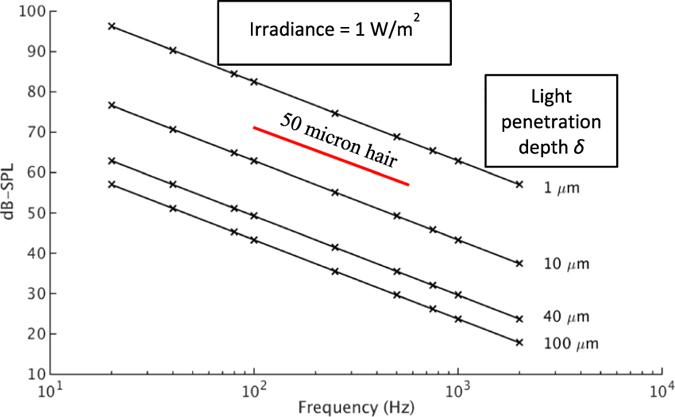
Simulation results for half-space illuminated by sinusoidally varying light flux and human hair (dashed line).

We calculate the air pressure fluctuation at the surface using the predicted surface temperature and the ideal gas assumption. We report the sound pressure levels near the solid’s surface without consideration of the distance to, or the geometry of, the transducer. These should be considered for actual applications. Figure 3 shows the SPL versus the frequency *f* for several light penetration depths δ as can be calculated using equation 4. A strong inverse dependence on both *f* and δ is apparent.

## Hair and Fibers

We also are interested in fibrous photoacoustic transducers, typified by hair and clothing. It seems significant that people with frizzy hair are reported to be more likely to hear concurrent sound from meteors^1^,^2^. Intuitively, frizzy hair should be a good transducer for two reasons. Hair near the ears will create localized sound pressure, so it is likely to be heard. Also, hair has a large surface-to-volume ratio which maximizes sound creation. The following paragraphs describe how we calculate the SPL generated by an individual hair illuminated by a sinusoidally varying light.

**Figure 4:**
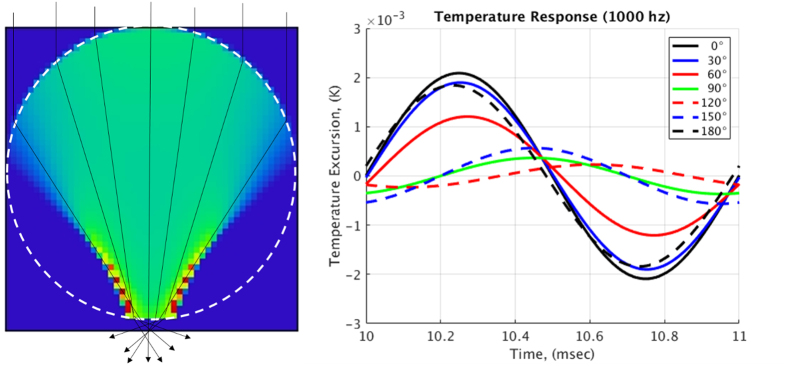
(**a**) Rays traced through a 50-μm diameter hair and distribution of energy absorbed within the hair. The green color indicates absorption equal to A≈I_0_ * 3%/μm. The yellow is twice this and red is 3 times as much. (**b**) For an incident flux of 1 W/m_2_ this is the computed variation in surface temperature relative to the mean at 30° intervals around the hair.

Figure 4a shows a fan of rays traced through a hair in addition to the volumetrically absorbed power. We developed a time-dependent finite element model of a hair to compute the temperature distribution caused by the absorbed light. The computed temperature profile for a light intensity of 1 W/m^2^ (versus ~0.01 w/m^2^ for a −12 brightness fireball) in the hair^19^,^20^,^21^ permits its surface temperature to be calculated at a number of points (Fig. 4b). The surface temperatures were spatially-averaged and used to calculate the SPL due to the hair (the dashed line in Fig. 3b). Note that the wavelength of the sound is far larger than the hair diameter, so each hair heated by a pulse of light will create a line of heated air and will show little directionality. Also, the sound from closely-spaced hairs will add coherently.

## Summary

Our experimental measurements of photoacoustic sound intensity for (Fig. 2) paint, wood, a synthetic brown wig, and several types of dark cloth compare well with the analytical results. The wood half-space model matches the experiment if the light penetration depth δ is 20 μm. The hair model differs slightly from the experiment. With an irradiance level of 1 W/m^2^, the wig’s measured SPL was 40 dB while the calculated value was 47 dB. A difference of 2 dB could be due to the differences in hair diameter: 50 μm for the model^20^ versus ~80 μm for the wig. Additionally, the wig is synthetic which could lead to a further difference of ~3 dB. These two corrections add to 5 dB which is similar to the 7 dB difference between modeling and experiment.

Our calculations and experiments are consistent with how observers have described the concurrent sounds associated with fireballs. This suggests that an observant person in a quiet environment containing good transduction materials could hear photo-acoustically induced sound from a −12 magnitude or brighter fireball—assuming it emits light modulated at acoustic frequencies.

Two final experiments were performed. The irradiance E(t) signal measured on Earth from fireball EN091214 was used to drive the white light source. The recorded photoacousticly generated sound, available in the supplementary section file “EN091214 & black paint transducer,” is similar to distant thunder, partially because the illumination was sampled at 5 kHz. The sound was clear but could also have been interpreted as noise, so to further satisfy our intuition, we drove the light source with the folk tune Greensleeves and recorded the photoacoustic sound. This is available in the supplementary section file “Greensleeves & black paint transducer”. The signal-to-noise was low, but one can easily identify the tune. Finally, we verified that electrical signals were not “leaking” into the microphone channel. The light source was rotated away from the sample, at which time the signal level dropped ~100X.

## Additional Information

**How to cite this article:** Spalding, R. *et al*. Photoacoustic Sounds from Meteors. *Sci. Rep.* **7**, 41251; doi: 10.1038/srep41251 (2017).

**Publisher's note:** Springer Nature remains neutral with regard to jurisdictional claims in published maps and institutional affiliations.

## Supplementary Material

Supplementary Section

Supplementary Video

## Figures and Tables

**Figure 1:**
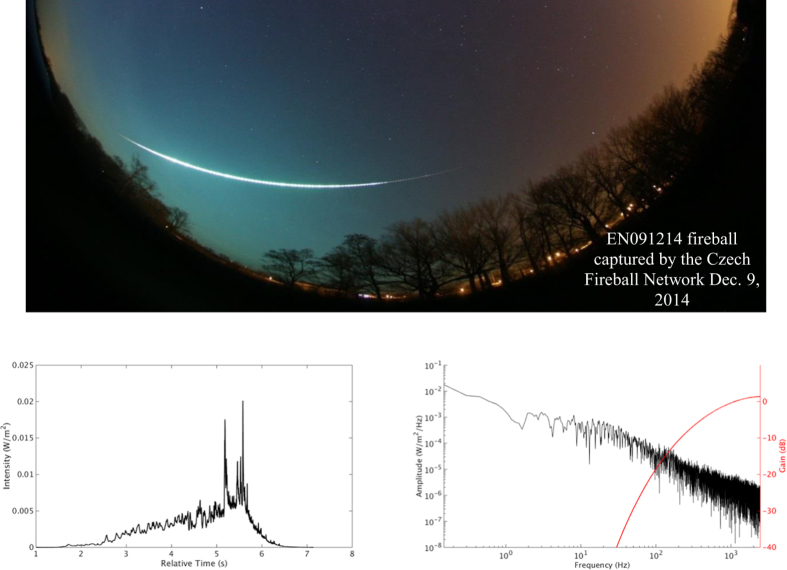
(**a**) Open-shutter photograph of fireball EN091214^18^ taken by Dr. Spurny (coauthor) at the Czech Fireball Network. (**b**) Intensity at a slant distance of 100 km for fireball EN091214 from Dr. Spurny. The CFN radiometers have flat response below 5 kHz. Intensity curves from different fireballs are in supplementary section. (**c**) Fourier transform of intensity-time history along with the normalized gain of the human ear for reference.
