# Supplementary material for: Photoacoustic Sounds from Meteors
Source: Sci Rep. 2017 Feb 1;7:41251. doi: 10.1038/srep41251 (PMC5286498; doi:10.1038/srep41251)
Supplement: Supplementary Section [file srep41251-s1.pdf]

Supplementary Section for "Photoacoustic Sounds from Meteors"

Richard Spalding[1], John Tencer[1], William Sweatt[1], Benjamin Conley[1], Roy Hogan[1], Mark Boslough[1]

GiGi Gonzales[1], & Pavel Spurný[2]

[1]Sandia National Laboratories, Albuquerque, NM, USA

[2] Astronomical Institute, Czech Academy of Sciences, Ondřejov, Czech Republic

1.) More references for concurrent sounds: In addition to the four references in the main text which describe observations of concurrent sounds, NASA has an article on their website that discusses concurrent sounds heard during the Leonid meteor shower on Nov. 18, 2001[S1]. NASA has a second website devoted to eye-(and ear-) witness accounts of Leonids 2001[S2].

2.) Intensity plots of several fireballs measured by the Czech Fireball Network. These are typical of all of the fireball intensity traces that we have seen. They each appear to be a slowly changing black body plus a series of closely-spaced intensity spikes.

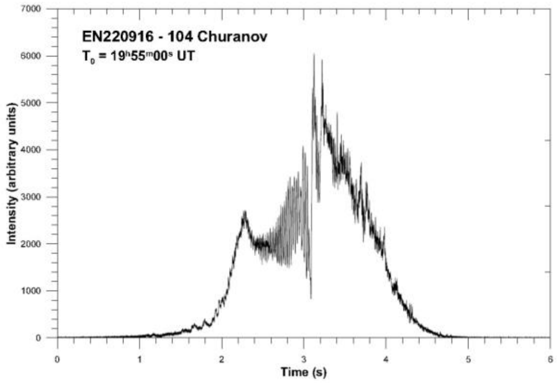

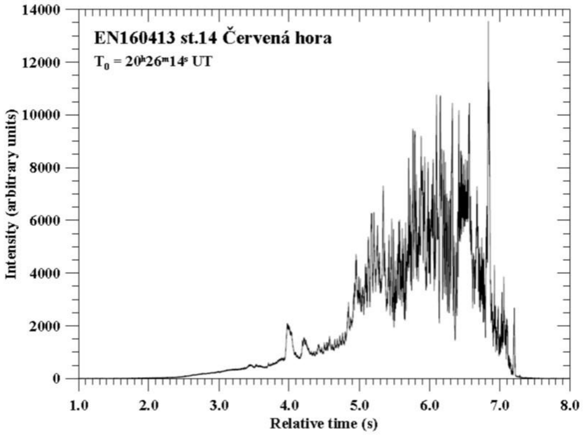

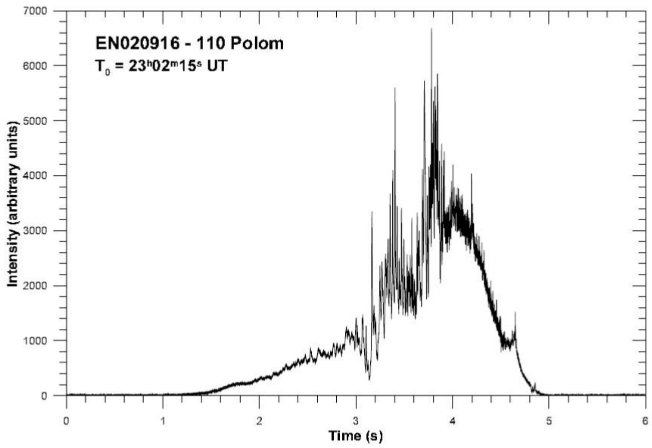

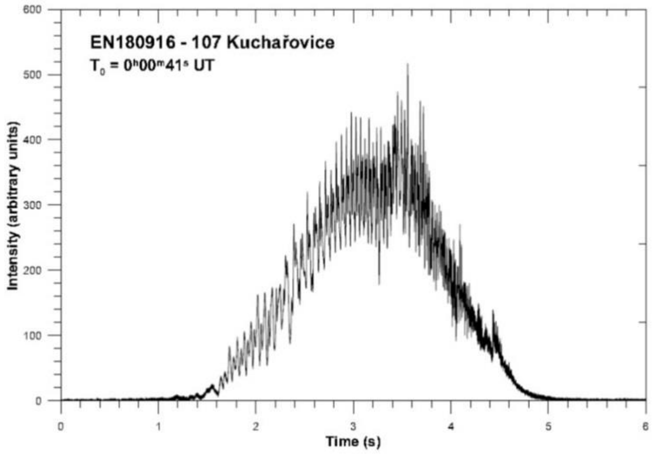

3.) The apparent magnitude of a star or other astronomical body is a measure of how bright it appears to a human. For further details see https://en.wikipedia.org/wiki/Apparent_magnitude.

4.) The fireballs can be composed of rock, dirty ice, and anything between. A magnitude -11 fireball can weigh between a few kg up to several 10's of kg, and can enter the atmosphere at velocities between ~10 and ~40 km/s.[S13]

5.) A. Rosencwaig and RJ Emary filed a US patent covering PAS, photoacoustic spectroscopy, US4129385[S3].

6.) SPL—the sensitivity of the human ear. There is an international standard describing human hearing sensitivity. It was developed by the International Electrotechnical Commission, numbered (IEC) 61672:2003[S4].

7.) The sound pressure level L_P=1 dB is for a sound intensity of 10^{-12} W/m^2.

8.) About the experiment: The experimental setup consisted of a 10 cm square, 1.7 W Commercial Electric-T41 SoftWhite LED array, the sample, and a Brüel & Kjær microphone, all inside an anechoic chamber. Outside we located an Agilent 33250A-Signal Generator plus Venable Instruments VLA1500 Linear Amplifier driving the LEDs at 1 kHz and an Agilent E4446A recording the sound spectrum.

A Plexiglas window with thick black Krylon paint on its front was also tested. The plate was turned around so the paint was on the rear face, and then it was retested. The resulting signal was approximately 10 dB less, indicating that PA is primarily a front-surface effect.

9.) Thermal properties of common dielectric materials: The average thermal properties for common dielectric materials can be found in hundreds of engineering and physics handbooks published by the ASME, AAP, CRC, and countless other sources. In general, dielectric materials show a wide range of material properties based on chemical composition. Furthermore, if the materials are porous like wood, leaves, or hair, the void space can range from very little to several tens of percent. For these reasons we have not tried to find original references. Instead we suggest the web reference "Engineering Toolbox."[S5]

For thermal properties of wood, we used the values for white pine: specific heat=1700 J/(kg-K), density=700 kg/m^{3,} and conductivity=0.12 W/(m-K)[S6].

Paint properties also vary widely for the many compositions including oil-based, water-based, and acrylic paints. We have offered an average value, knowing that one can encounter a wide variation of the properties[S7].

Properties of hair are in the literature: VanKampen[S12] measured hair's refractive index, n= 1.56, and the absorption depth of black hair, $\delta \approx$cm/150. The thermal diffusivity of leather (listed above) is similar to that of hair: D=0.07 μW/(^{0}K*m^2). The diameter of fine hair[S12] is $d_{hair} \approx 50$ µm.

10.) The half-space problem assumes that the temperature response of the solid sample material is independent of the temperature response of the adjacent air due to the large dissimilarity in their thermal conductivities and heat capacities. This assumption was supported by initially simulating both domains and then later restricting the simulation to only the solid with the interaction at the interface modeled using a Robin boundary condition. The temperature fluctuation at the interface surface was then used as a Dirichlet boundary condition when simulating the air. The pressure and temperature of

the air was assumed to follow the ideal gas assumption. The air is assumed to be transparent to the incident radiation which itself is a collimated beam. Attenuation within the solid is assumed to be exponential consistent with a purely absorbing media although the magnitude of the resulting surface temperature fluctuation was observed to be insensitive to the scattering albedo.

11.) Robin boundary condition: A Robin boundary condition is a weighted combination of Dirichlet and Neumann boundary conditions. The Robin boundary condition is called a convective boundary condition in heat transfer problems[S10].

12.) Developing the SPL for hair: We employed ZEMAX, a widely-used commercial optical design and analysis computer code, to trace the rays through a hair and then compute the flux absorbed in the hair[S8]. ZEMAX calculates the heat deposition distribution [$\varphi(r,\theta)$] within a hair. This must be multiplied by the temporal variation of the illumination from the bolide to produce the thermal input for a hair.

"Frizzy hair" typically implies dry, small diameter hair in tight curls, often covering the ears.

For computing the photoacoustic response of hair, it is simplest to work in frequency space. The light signal from a bolide can be transformed into a Fourier series in time, $E(t) = \Sigma[K_n * \sin(f_n t)]$. A frequency-based output is convenient because the sensitivity of the human ear is described in frequency space. For one Fourier term of the heat input [$K_n \sin(f_n t)$], the instantaneous flux input is $\Phi(r,\theta,t)=\varphi(r,\theta) * K_n * \sin(\omega t)$. This is the heat input, and from it we must calculate the heat flow within the hair which allows the temperature distribution as a function of time to be computed.

A widely used finite-element computer code was used to calculate the time-varying temperature field within the hair due to the heat input $\Phi(r,\theta,t)$.[S9]

It is interesting that the temperature peaks and valleys reach the different points on the surface at different times. When the components are summed, this phase has to be taken into account.

13.) Material properties of hair: As stated in the main paper, the thermal properties of hair probably are similar to epidermis which is similar to leather— for which there are values available[S5]. Optical properties, refractive index, and absorption are presented in van Kampen's MS thesis[S11]. Hair diameter ranges from about 50 µm to 70 µm[S12]. We chose the smaller value to represent "frizzy hair."

14.) Discussion of results: There is a 7 dB discrepancy between the sound level calculated for the real hair and that measured for the wig. Several things contribute to the

inconsistency. First, the hair diameter was assumed to be 50 µm while the diameter for the wig hair was 80µm. This gives a surface area-to-volume ratio of 1.6 which could, crudely speaking, produce a 2 dB difference in sound level. Second, the thermal diffusivities of real hair and the synthetic hair are 0.07µW/(Km^2) and ~0.18µW/(Km^2). The square root of this ratio is 2.1, giving a 3 dB difference. These two correction factors add to 5 dB which is close to the 7 dB difference between the calculated and measured SPL levels.

15.) Actual sound from the photoacoustic testing: The optical signal recorded from fireball EN091214 was used to drive the light source. The sound recorded during this test was not very compelling. (It's white noise after all.)

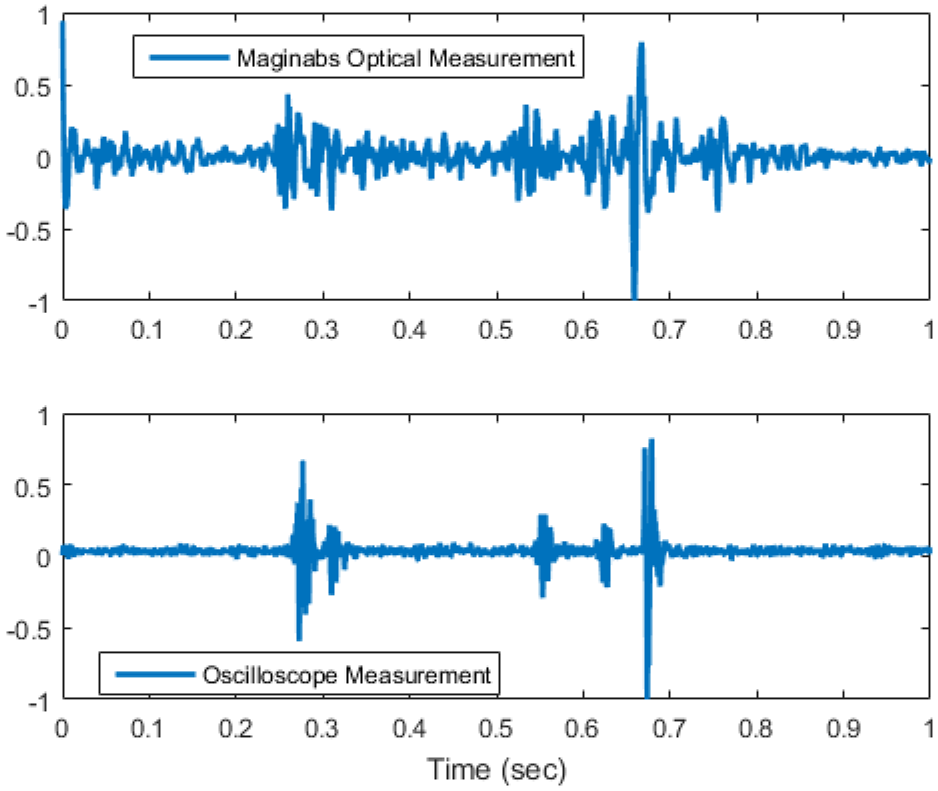

To try to convince ourselves that the PA effect was real, BC played an English folk tune ("Greensleeves") on his violin which we used as a signal to drive the light source. The output sound captured by the microphone was quite noisy, but one definitely can make out the tune. Listen in stereo if possible.

To hear Greensleeves, download supplementary data set

16.) Thanks to Michael Sinclair (SNL) who alerted us to photo-acoustic spectroscopy and Andreas Schmitt-Sody for critical review.

Further References:

[S1]NASA, 2001a," Listening to Leonids", *NASA Science News*, Nov 26, 2001, http://science.nasa.gov/science-news/science-at-nasa/2001/ast26nov_1/. Date of access: 01/06/2016

[S2]NASA,2001b, "Leonid Meteor Sounds, Nov 18, 2001, http://www.spaceweather.com/meteors/leonids/2001/sounds.html Date of access: 01/06/2016

[S3] Rosencwaig, A. & Emary, RJ, *Photoacoustic Sample Cell,* United States Patent US4,129,385 Dec. 12, 1978

[S4]See English Wikipedia version (original author Skirrow PJ (2007)) at: http://en.wikipedia.org/wiki/A-weighting or the Commission's original report by the Commission Electro-technique International in French; https://webstore.iec.ch/preview/info_iec61672-1%7Bed2.0%7Db.pdf. Date of access: 01/06/2016

[S5]"Engineering Toolbox, 2014"; http://www.engineeringtoolbox.com/. Date of access: 01/06/2016

[S6]Incropera, Frank P. & et.al, "Fundamentals of Heat and Mass Transfer", 6th ed., John Wiley, 2006.

[S7]Raghu, O & Philip, J, "Thermal properties of paint coating on different backings using a scanning photo acoustic technique." *Measurement Science and Technology*, 17, 2006, p2945-2949.

[s8]ZEMAX which is owned and distributed by ZEMAX-LLC, 22908NE Alder Crest Drive, Suite 100, Redmond, WA 98053.

[S9] Gartling, D.K., Hogan, R.E., & Glass, M.W. "Coyote – A Finite Element Computed Program for Nonlinear Heat Conduction Problems", SAND2010-0714. It is available from Sandia National Laboratories, Albuquerque, NM. (2009)

[S10] Hahn, David W.; Ozisk, M. N. *Heat Conduction, 3rd edition*. New York: Wiley. ISBN 978-0-470-90293-6), (2012).

[S11] vanKampen, Tjitske, 1997, "Optical Properties of Hair", MS thesis, Tech. U, Eindhoven

[S12] Franbourg, et al. "Current research on ethnic hair" *J. Am. Acad. Dermatology,* June 2003, p. S115

[S13] Z.Ceplecha, R. Spalding, Jacobs, c. & E. Tagliaferri, "Luminous efficiencies of bolides", *SPIE* Vol. 2813, 46-56 (1996)
